# Only anti-GM4 antibody positivity in a Chinese girl with overlapping MFS/GBS: a case report

**DOI:** 10.1007/s10072-024-07300-6

**Published:** 2024-01-25

**Authors:** Jing Chen, Maoqiang Tian, XiaoMei Shu

**Affiliations:** Department of Pediatrics, Affiliated Hospital of Zunyi Medical University, No. 143, Dalian Road, Zunyi, 563003 China

**Keywords:** Guillain-Barré syndrome, Miller-Fisher syndrome, Overlapping MFS/GBS, Anti-GM4 antibodies

## Abstract

**Background:**

Guillain-Barré syndrome (GBS), as the most common cause of acute flaccid paralysis worldwide, is considered a part of a clinical spectrum in which discrete, complete, or incomplete forms of GBS and overlapping syndromes lie on the basis of their clinical features. The term overlapping Miller Fisher syndrome (MFS)/GBS is used when patients with MFS also suffer from progressive motor weakness of the limbs. Anti-ganglioside GQ1b has been specifically associated with MFS and ophthalmoplegia.

**Case description:**

Here, we report a Chinese girl who was diagnosed with overlapping MFS/GBS showing acute flaccid paralysis of all four limbs, sensory symptoms, cranial nerve dysfunction, autonomic involvement, ophthalmoplegia, and ataxia. She had high serum and cerebrospinal fluid titres of monospecific anti-GM4 IgG antibody instead of anti-GQ1b antibody in the acute phase.

**Conclusion:**

Anti-GM4 antibodies usually coexist with other antiganglioside antibodies, leading to missed diagnoses. The findings of the present study show that antibodies to ganglioside GM4 may in overlapping MFS/GBS as the lone immunological factors.

## Introduction

Miller-Fisher syndrome (MFS), a clinical variant of Guillain-Barré syndrome (GBS) with a frequency that varies from 5 to 35%, is characterized by ophthalmoplegia, ataxia, and areflexia.

Fifty percent of MFS patients may present with overlapping features of other GBS variants, which are defined as MFS/overlap variants, including MFS/GBS, Bickerstaff’s brainstem encephalitis (MFS/BBE), and pharyngeal-cervical-brachial (PCB/MFS) variant [1]. Anti-GQ1b immunoglobulins, which are antiganglioside antibodies (AGAbs), are found in 85–95% MFS patients. GM4, a less-frequently discussed monosialic ganglioside, is found in high amounts in myelin and to a lesser extent in astrocytes. Anti-GM4 antibodies are usually detected in combination with other subtypes of antiganglioside autoantibodies. Here, we report the case of a Chinese girl with overlapping MFS/GBS, who had only anti-GM4 IgG antibodies.

## Case presentation

**Fig. 1:**
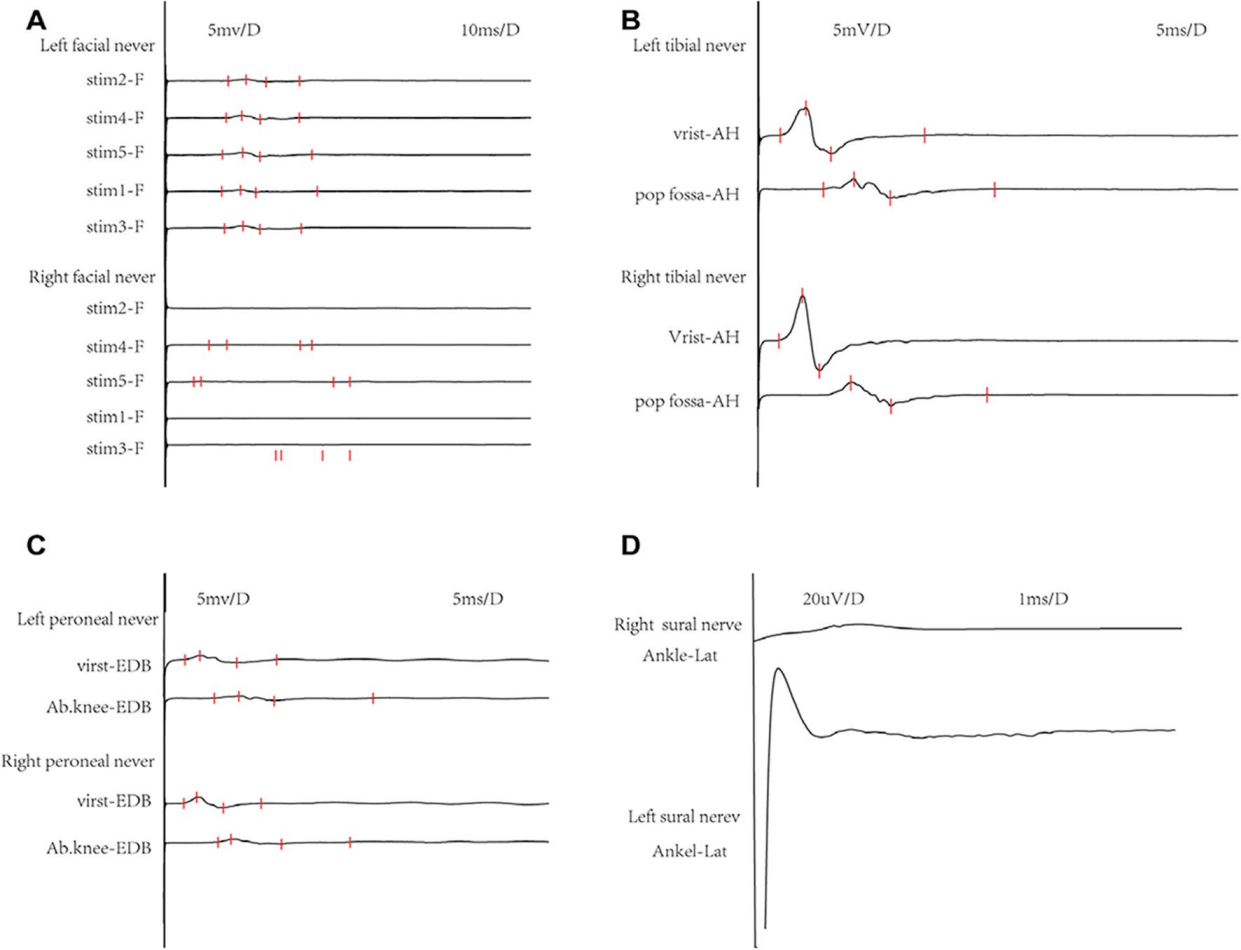
Nerve conduction studies of the patient. The absence of compound muscle action potentials (CMAP) in right the facial nerve, the amplitude of the distal CMAPs was obviously decayed in the left facial nerve (**A**); the bilateral tibial and common fibular nerves, the CMAP amplitude was reduced and the motor nerve conduction velocity (m-NCV) was slowed (**B, C**); sensory nerve action potentials (SNAPs) could not be detected in the common fibular nerve (**D**)

A Chinese 3-year-old girl, who had normal intelligence and an unremarkable medical history and family history, was hospitalized in June 2022 because she was suffering from the terrible pain in the lower limbs and abnormal gait for 4 days. She had no recent vaccination, obvious digestive, or respiratory symptoms before the onset of the symptoms. On admission, physical examinations showed positivity for the following main nervous system signs: nuchal rigidity; bilateral ptosis; facial nerve weakness (showing complete disappearances of frontal wrinkles and a decrease depth of the bilateral nasolabial sulcus), which was slightly more prominent on the right side; hand tremors and severe truncal ataxia resulting in poor balance and head control; low muscular tension; areflexia in all limbs; and Romberg’s sign. The muscle strength of the bilateral distal and proximal limbs was grade II and grade III, respectively. In the hospital for 5 days, she had hoarseness, ptyalism, and dysphagia, with no influence on slowly drinking and eating fluid food. In the 11 days of her illness, her condition becomes worse, with undergoing urinary and fecal incontinence and excessive sweating. The findings of cranial MRI and cervical and thoracic spinal MRI were normal. Early in the disease course, nerve conduction studies (NCS) findings showed that the absence of compound muscle action potentials (CMAP) in the right facial nerve, the amplitude of the distal CMAPs was obviously decayed in the left facial nerve, and the bilateral tibial and common fibular nerves, the CMAP amplitude was reduced, the motor nerve conduction velocity (m-NCV) was slowed, sensory nerve action potentials (SNAPs) could not be detected in the common fibular nerve, and F-wave latency and H-wave absence were observed (Table 1; Fig. 1). Lumbar puncture was performed 1 week after onset, and cerebrospinal fluid (CSF) analysis showed that the protein level was 1.2 g/L (normal values 0.2–0.4 g/L), while the white blood cell count was 3 × 10^6^/L (normal values 0–5 × 10^6^/L). Her acute-phase serum and CSF samples were investigated by enzyme-linked immunosorbent assay for anti-ganglioside antibodies (for sulfatides, GM1, GM2, GM3, GM4, GD1a, GD1b, GD2, GD3, GT1a, GT1b, and GQ1b), including IgG and IgM. Only anti-GM4 IgG antibodies were detected, and the other 11 antigen tests had negative results. A clinical diagnosis of overlapping MFS/GBS was made based on the patient’s clinical presentation. Intravenous immunoglobulin (IVIG) therapy at the dosage of 2.0 g/kg was immediately initiated on the seventh day of the course of disease, followed by rehabilitation therapy. The neurological symptoms were gradually resolved by 1 month from the onset of symptoms in the sequence of neuropathic pain, ataxia, autonomic dysfunction, cranial nerve palsy, peripheral nerve paralysis. During an ill period of 3 months, her motor strength was 4/5 bilaterally in all limbs, with a complete improvement in her multiple cranial nerve palsies.

**Table 1:** Nerve conduction studies (NCS): motor nerve conduction parameters

	Distal latency (ms)	Amplitude (mv)	MCV (m/s)	*F*-wave (ms)
Left peroneal nerve	Ankle-EDB 5.54	Ankle-EDB 0.54		
	Ab.knee-Ankle 13.1	Ab.knee-Ankle 0.29	Ab.knee-Ankle 19.2	38.4
Right peroneal nerve	Ankle-EDB 5.21	Ankle-EDB 0.83		
	Ab.knee-Ankle 14.1	Ab.knee-Ankle 0.37	Ab.knee-Ankle 17.4	43.5
Left tibial nerve	Ankle-AH 4.71	Ankle-AH 2.6		
	Pop fossa-Ankle 13.7	Pop fossa-Ankle 0.99	Pop fossa-Ankle 22.2	
Right tibial nerve	Ankle-AH 4.42	Ankle-AH 4.3		
	Pop fossa-Ankle 15.0	Pop fossa-Ankle 1.15	Pop fossa-Ankle 18.0	
Left facial nerve	Stim1-Frontalis 15.6	Stim1-Frontalis 0.16		
	Stim2-Frontalis 17.3	Stim2-Frontalis 0.2		
	Stim3-Frontalis 16.3	Stim3-Frontalis 0.28		
	Stim4-Frontalis 16.8	Stim4-Frontalis 0.29		
	Stim5-Frontalis 15.8	Stim5-Frontalis 0.25		

*MCV* motor conduction velocity, *F-wave F*-wave latency, *EDB* extensor digitorum brevis, *Ab.knee* above knee, *AH* adductor hallucis, *Pop fossa* popliteal fossa, *Stim* stimulate

## Discussion

GBS is an inflammatory polyneuropathy characterized by progressive flaccid ascending and symmetric muscle weakness associated with motor features and the presence or absence of sensory symptoms [2]. GBS is subclassified into classic forms, which present with acute flaccid paralysis of all four limbs, and localized forms, including pharyngeal–cervical–brachial weakness, acute pharyngeal weakness, paraparetic GBS, and bifacial weakness with paresthesias. MFS is characterized by the symptom triad of ophthalmoplegia, ataxia, and areflexia and is divided into classic, CNS subtypes, incomplete forms consisting of acute ophthalmoparesis, acute ataxic neuropathy, acute ptosis, and acute mydriasis [3]. The classic forms of GBS and MFS and their subtypes constitute the GBS spectrum and overlapping syndromes, which share common clinical features, including a history of previous infection, a single-phase course, symmetry, cranial or limbs weakness, CSF albumin cell separation (high protein and normal cell count), AGAbs, and evidence of demyelinating neuropathy neurophysiology. Overlapping MFS/GBS can be considered when patients suffer from progressive motor weakness of the limbs at the same time or subsequently and account for approximately 15−76.2% of MFS/overlap variants [1]. In this case, the patient had an acute onset of symptoms, including acute flaccid paralysis of all four limbs, sensory symptoms, weakness of posterior cranial nerves VII and IX, ophthalmoplegia, ataxia, and areflexia at the same time. The clinical manifestation was characterized by a slowly progressive course with autonomic involvement. Albumin cytological dissociation and anti-GM4 antibodies were observed in the CSF 11 days after onset. The NCS findings met the Hadden’s criteria for acute motor sensory axonal neuropathy (AMSAN). Therefore, the patient was diagnosed with the incomplete overlapping MFS/GBS.

GBS is often (up to 70%) preceded by a respiratory or gastrointestinal tract illness due to infections with various pathogens, such as *Campylobacter jejuni*, Epstein–Barr virus, influenza virus, cytomegalovirus, *Mycoplasma pneumoniae*, enteroviruses, and the severe acute respiratory distress syndrome–coronavirus-2 (SARS-CoV-2) which is increasingly being reported as a prodromal infectious agent of GBS over the past few years [4, 5]. Other less common precipitants are surgery, pregnancy, cancer, and vaccinations. In the present case, the trigger for overlapping MFS/GBS was unclear in the absence of obvious digestive symptoms or respiratory symptoms within 1 to 2 weeks before the onset of the symptoms and testing for pathogens. However, this does not rule out the existence of a preceding infection that is mild and sometimes minimized or forgotten by patients [4].

The immunological pathogenesis of GBS involves antibody attack of a variety of target glycolipid antigens on nerve terminals and axons. In the acute phase of GBS, AGAb titres are frequently elevated and may be associated with specific clinical features in that the distribution of the damage by binding to the respective ganglioside antigens has a unique localization [6]. These antibodies are useful diagnostic markers and possible pathogenetic factors [7]. In particular, IgG anti-GQ1b antibodies are positive in as many as 90% of patients with MFS [8]. Few studies have addressed the AGAbs of overlapping MFS/GBS, most of which are case reports or small studies. Sekiguchi et al. indicated that the IgG of anti-GQ1b, anti-GM1, and/or anti-GD1a existed in 73.9% (17/23) and in 21% (5/23) of overlapping MFS/GBS patients, respectively, in a study with a sample size of 33 [9]. A study from the Lisette Bazán-Rodríguez group showed that anti-GQ1b antibodies were positive in 60% (2/4) of patients with overlapping MFS/GBS [1]. Rinsho Shinkeigaku reported the case of a 70-year-old woman suffering from marked oculomotor nerve disturbance with ganglioside complexes (GSCs), including IgG antibodies against GM1, GM1b, and GD1a without anti-GQ1b IgG antibody [10]. Caudie reported that a patient who developed the overlapping sensory ataxic form of GBS and MFS had high titres of monospecific anti-GD1b IgG antibody following *C*. *jejuni* infection and concluded that antibody to GD1b ganglioside is one of the immunological factors in the pathogenesis of the sensory ataxic form of GBS [11]. Furthermore, some patients with MFS/GBS are negative for AGAbs [12, 13].

GM4, as the predominant ganglioside species, not only is specifically localized in the myelin of the CNS but also is present in oligodendroglia and neurons [14]. In 2007, anti-GM4 antibodies for the first time were detected in the patients of peripheral neuropathy (PNPs), including GBS, MFS, and multifocal motor neuropathy (MMN) in combination with other AGAbs, and in the patients of unclassified PNPs as the only antibody [15]. Yoshikawa et al. discovered that anti-GM4 antibodies and anti-GD1b antibodies coexist in BBE and GBS with ophthalmoplegia (GBS-OP) [16]. A recent study showed that the addition of fluorinated GM4 to oligodendrocyte progenitor cells in vitro increased the differentiation of mature oligodendrocytes, and the manipulation of GM4 could influence myelination in therapeutic strategies [17].

GM4, as the predominant ganglioside species of myelin with GM1, is less frequently discussed. Anti-GM4 antibodies have attracted little attention, perhaps because anti-GM4 antibodies mostly coexist with other AGAbs in PNPs. In addition, most AGAb detection methods do not detect GM4, leading to a decrease in the positive rate. The patient whose case is reported here was diagnosed with overlapping MFS/GBS and had high titres of monospecific anti-GM4 IgG antibody in serum and CFS without other AGAbs that have been reported in previous studies on overlapping MFS/GBS. It is likely that antibodies against GM4 are associated with overlapping MFS/GBS as the main and only immunological factors. Of course, additional studies especially focusing on the pathogenesis of GM4 in GBS are needed to understand the clinical feature of this condition.

Overlapping MFS/GBS has a good recovery profile and no significant severe complications when IVIG is initiated immediately. Most patients improve within 1 to 2 months and make a complete recovery in 6 months without specific treatment [18]. The specificity between AGAbs and prognosis has not been discovered [12].

## Conclusion

We believe that antibodies against ganglioside GM4 play an important role in GBS, which has yet to be proposed thus far. More attention should be given to overlapping MFS/GBS related to GM4.
